# Differences in Processing Quality Traits, Protein Content and Composition between Spelt and Bread Wheat Genotypes Grown under Conventional and Organic Production

**DOI:** 10.3390/foods10010156

**Published:** 2021-01-13

**Authors:** Verica Takač, Viola Tóth, Marianna Rakszegi, Sanja Mikić, Milan Mirosavljević, Ankica Kondić-Špika

**Affiliations:** 1 Institute of Field and Vegetable Crops, Maksima Gorkog 30, 21000 Novi Sad, Serbia; sanja.mikic@ifvcns.ns.ac.rs (S.M.); milan.mirosavljevic@ifvcns.ns.ac.rs (M.M.); ankica.spika@ifvcns.ns.ac.rs (A.K.-Š.); 2 Centre for Agricultural Research, Agricultural Institute, Brunszvik u. 2, 2462 Martonvásár, Hungary; toth.viola@atk.hu (V.T.); rakszegi.mariann@atk.hu (M.R.)

**Keywords:** bread wheat, conventional, gliadin, gluten, glutenin, grain, organic, protein, quality, spelt

## Abstract

The unique rheological properties of bread wheat dough and the breadmaking quality of its flour are the main factors responsible for the global distribution and utilization of wheat. Recently, interest in the production and expansion of spelt wheat has been boosted due to its significance in the production of healthy food, mostly originated from organic production. The aim of this study was to examine and compare quality parameters (gluten content, Zeleny sedimentation volume, farinograph dough properties), protein content and composition (by the Dumas method, Size Exclusion (SE) and Reversed Phase (RP) High Performance Liquid Chromatography (HPLC) analyses) of five bread and five spelt wheat varieties grown under conventional and organic production in Hungary and under conventional production in Serbia. Most of the analyzed traits showed significant differences between varieties, wheat species and growing sites. Total protein content was significantly higher in spelt than in bread wheat and under conventional than under organic production. In comparison to spelt, bread wheat showed better breadmaking quality, characterized by a higher amount of glutenins (in particular high molecular weight glutenin subunits) and unextractable polymeric proteins. The proportion of the gliadins was also found to be different under conventional and organic systems. Spelt Ostro and Oberkulmer-Rotkorn and bread wheat varieties Balkan, Estevan and Pobeda proved suitable for low input and organic systems.

## 1. Introduction

Wheat (*Triticum* sp.), as one of the most important cultivated crops, represents staple food for the majority of the human population. Its range of cultivation includes all inhabited continents, on different longitudes, latitudes and altitudes, encompassing diverse environmental conditions. Among all *Triticum* species, bread wheat (*Triticum aestivum* L. subsp. *aestivum*, 2n = 6x = 42, genome AABBDD, where “n” is the ploidy level and “2n” refers to diploid genome where each cell contain two copies of each chromosome. The “x” refers to the total number of chromosomes in haploid genome and “6x” refers to overall number of chromosomes originated from three independent haploid genomes) is the most widely grown. In the last few decades, more attention is oriented to the production of spelt wheat (*T. aestivum* L. subsp. *spelta*, 2n = 6x = 42, genome AABBDD) and its use in various diet products [1]. Although bread and spelt wheat have the same genome (AABBDD), they differ in some important traits. Spelt plants are taller, have longer ears, a brittle rachis and glumes tightly adhered to the seed, protecting it from dispersion and against various pathogens, insects, birds and rodents [1,2]. Spelt wheat was one of the first cultivated crops [1]. It was widely cultivated until the last century. From the beginning of the 20th century, the cultivation of spelt wheat declined and was suppressed by higher-yielding, free-threshing bread wheat varieties [1]. Recently, a new interest for production and expansion of spelt wheat have been appeared due to its significance in the production of healthy food products [3], mostly originated from organic fields, due to its capability to grow at organic and low input fields [4], higher protein [5,6] and gluten content in comparison to bread wheat [7].

The unique rheological properties of bread wheat dough and the breadmaking quality of its flour are the main factors determining the global distribution and utilization of wheat. Wheat flour quality mostly depends on composition and the quantity of wheat proteins, mostly gluten proteins [8,9]. Wheat proteins present in the embryo, endosperm and aleurone layer of the grain, consist of gluten and non-gluten proteins. The gluten proteins account for 85% of the total grain proteins, encompassing polymeric glutenins and monomeric gliadins. According to the molecular weight distribution, polymeric glutenins are classified into high molecular weight (70,000–90,000 Da) and low molecular weight (20,000–45,000 Da) glutenin subunits (High Molecular Weight Glutenin Subunits—HMW-GS and Low Molecular Weight Glutenin Subunits—LMW-GS, respectively), while monomeric gliadins are classified as α/β, γ and ω gliadins, based on the order of mobility on electrophoresis at low pH. Albumins and globulins are considered non-gluten proteins. Glutenin macropolymers are also known as unextractable polymeric proteins (UPP) or insoluble glutenins, while smaller size glutenin polymers are known as extractable polymeric proteins (EPP), or soluble glutenins, based on the extractability of grain protein fractions in the sodium dodecyl sulfate (SDS) extraction buffer. Wheat quality is a result of high polymorphism found in storage proteins [10]. Gluten proteins are responsible for processing the quality of wheat dough; in particular, glutenins contribute to its strength and elasticity, whereas gliadins for its extensibility [11]. Among glutenins, HMW-GS are one of the main quality determinant responsible for strength and elastic properties of dough and is encoded by polymorphic genes at *Glu-1* loci (*Glu-A1*, *Glu-B1* and *Glu-D1*) present on the long arms of the group 1 chromosomes [12]. Equitably crucial for flour quality, favorable rheological properties of dough and hence the quality of the end-use products are the relative proportion of gliadins and glutenins and the relative amount of large insoluble glutenin polymers [12]. Much higher content of total glutenins and lower content of total gliadins and gliadin to glutenin ratio found in bread wheat in comparison to spelt wheat gave unique rheological properties and good breadmaking quality of bread wheat dough [9]. Although all breadmaking traits are under genetic control, environmental conditions, management systems, the genotype by environment interactions, and the genotype by environment by management interactions very clearly modify grain quality, especially protein and gluten content [13,14].

Organic production is an agricultural management system that preserves the soil, plants and ecosystem in their natural state. It is based on the application of non-synthetic naturally occurring pesticides and fertilizers of organic origin. Despite lower yields, especially in wheat production [15], the benefits from organic production are various, such as improved physical and chemical properties of soil, enhanced crop diversity and biodiversity of beneficial insects and other micro and macrofauna, reduced pollution, environment preservation and protection [16], and likely positive effect on human health [17]. According to the Food and Agriculture Organization of the United Nations (FAO), there is a modest increase in area under organic crop production worldwide in the last ten years [18], while in the same period, in the countries of the European Union (EU), the area under organic crop production has risen by 70% [19]. In 2017, cereals were grown in 16% of the area under organic production in the EU, while in 2018, cereals accounted for 22% of the crops exported from the EU [19]. Interest in organic crop production in both Serbia and Hungary is expanding. Since 2012, the total area under organic production and specifically organic cereals in both countries have risen [20].

The effect of organic management on wheat protein content and breadmaking quality investigated in different studies were incongruent. Some authors reported that different management systems did not affect wheat protein content and breadmaking quality [15,21], whereas some findings showed that the protein content of bread wheat flour was lower in organic production in comparison to conventional [22,23]. Investigations on the differences in physical properties of seeds and processing quality traits between wheat species and varieties grown under organic and/or conventional management systems showed that the environment, management system and genotype had a strong significant influence on the physical properties of the grain [14,24].

Comparison of baking quality parameters between spelt and bread wheat genotypes under conventional management [25] and quality parameters between bread wheat genotypes under organic production was carried out in Hungary before. In Serbia, more attention was allocated to research on morphological and yield components [26] and breadmaking performance [27] of organically grown spelt wheat, as well as characterization of proteins from bread and durum wheat genotypes under conventional system [28]. However, little data are available on the comparison of quality parameters of bread and spelt wheat genotypes grown under both conventional and organic management systems in the conditions of the Pannonian Plain. Therefore, the aim of this study was to investigate the protein and gluten content and composition, and the processing quality traits related to them in different bread and spelt wheat varieties grown under distinct environmental and management conditions (organic, conventional) of Serbia and Hungary.

## 2. Materials and Methods

### 2.1. Plant Material and Field Experiment

The experimental material consisted of five spelt wheat and five bread wheat varieties originated from five European countries, namely Belgium, France, Germany, Serbia and Switzerland, mostly released during the 20th century (Table 1). The varieties were grown during the 2018/2019 season under conventional production in Serbia, at the experimental field at Institute of Field and Vegetable Crops, Novi Sad and under conventional and organic production in Hungary, in the field at the Center for Agricultural Research, Martonvásár.

In Serbia, the varieties were sown in 5 m^2^ plots in a randomized complete block design with three replications. The soil was of the chernozem type with soybean as a previous crop (Table 2). During the growing season, fertilizer was applied before sowing at the average dose of ca. 50 kg N/ha, 60 kg P/ha and 60 kg K/ha. In early February, an additional 50 kg N/ha of ammonium-nitrate (33% N) was top-dressed according to N-min analysis. In April, the plots were treated with herbicides (25 g/ha Stockstar (Stockton Chemical, Stockton, CA, USA) containing 500 g/ha tribenuron-methyl and 0.6 L/ha Lodin EC (HELM AG, Hamburg, Germany) containing 360 g/L fluroxypyr) and an insecticide (50 mL/ha Vantex CS (FMC Corporation, Philadelphia, Pennsylvania, United States) containing 60 g/L gama-cyhalothrin). In May, the treatment with gama-cyhalothrin was repeated, with an addition of a fungicide (1 L/ha Prosaro 250 EC (Bayer CropScience, Monheim am Rhein, North Rhine-Westphalia, Germany) containing 125 g/L tebuconazole + 125 g/L prothioconazole). Weeds from the plots were periodically hand-removed. Meteorological conditions for the 2018/2019 growing season (Table 3) were typical for Serbia.

Spelt and bread wheat varieties were sown in two replicate plots in the field at the Center for Agricultural Research in Martonvásár, Hungary. The plots were 2 m long, with six rows spaced at a 20 cm distance. The soil was of the chernozem type with a loam texture and pH 6.8–7.2. The previous crop was oil radish at the conventional site and phacelia at the organic site. The quantity of precipitation was a bit below the average, with a higher amount falling in the last 100 days before harvest. The temperature was typical in Hungary during the 2018/2019 season (Table 3).

Yearly average N input through NPK complex fertilizer was 120 kg/ha active ingredients at the conventional site. The nutrient supply of the organic field was assured by the previous crop. The plots were treated with herbicide (4 L/ha *U*-46 D-fluid SL (NUFARM, Cologne, Germany) containing 500 g/L 2-methyl-4-chlorophenoxyacetic acid; 40 g/ha Granstar 50 SX (FMC Corporation, Philadelphia, Pennsylvania, United States) containing 50% tribenuron methyl), insecticide (0.2 L/ha Karate Zeon 5CS (Syngenta, Basel, Switzerland) containing 50 g/L k-cihalotrin) and fungicide (first: 1 L/ha Amistar Extra (Syngenta, Basel, Switzerland) containing 200 g/L azoxistrobin and 80 g/L ciprokonazol, second: 1 L/ha Cherokee (Syngenta, Basel, Switzerland) containing 50 g/L ciprokonazol, 62 g/L propiconazol and 375 g/L cloretalonil) at the conventional site. No herbicides, insecticides or fungicides were used at the organic site.

### 2.2. Quality Parameters of the Grain and Flour

From the harvested samples, thousand-kernel weight (TKW) was determined with a Marvin System according to the standard MSZ 6367/4-86 (1986) method. Samples were milled on Perten 3100 Laboratory Mill (Perten, Hamburg, Germany) to produce whole meal, while after conditioning the grain to 15.5% moisture content Chopin CD1 (CHOPIN technologies, Villeneuve-la-Garenne, France) was used for the production of the white flour, containing only starchy endosperm. The total protein content was determined by the Dumas method according to AACC 46-30.01 method [39], with Elementar Rapid N III Analyzer (Elementar, Langenselbold, Hesse, Germany). The wet gluten content and gluten index (GI) were determined using a Glutomatic 2200 instrument (ICC 137/1, 155) (Perten, Hamburg, Germany). A Brabender farinograph (ICC 115/1) (Brabender, Duisburg, North Rhine-Westphalia, Germany) was used to determine the flour water absorption, dough stability and quality number. The Zeleny sedimentation test was carried out according to the standard ICC 116/1 method by using the SediCom System (developed at BUTE and produced by LabIntern Ltd., Budapest, Hungary) [40].

### 2.3. Protein Analysis

In order to determine the quantitative ratio of gluten proteins, the glutenins and the gliadins (Glu/Gli) and the amount of unextractable polymeric proteins (UPP), size exclusion high-performance liquid chromatography (SE-HPLC) was used. Every sample was analyzed in three replications. Sample preparation consisted of measuring 3 × 10 mg of the flour for total, soluble and insoluble fractions of gluten proteins. The flour was suspended in 1 mL 0.5% (*w/v*) SDS in phosphate buffer (pH 6.9) and sonicated for 15 s, for a fraction of total proteins, and 30 s for a fraction of insoluble proteins. Suspension of flour and SDS buffer for a fraction of soluble proteins was shaken on a laboratory shaker for 30 min. After sonication and shaking, samples were centrifuged for 10 min at 14,000 rpm, and the supernatant was filtered on a 0.45 µm polyvinylidene fluoride filter. Analyses were performed by The Waters Alliance™ HPLC System (Waters Corporation Milford, MA, USA) with 2695 Separation Unit and 2996 Photodiode Array Detector (Waters Corporation Milford, MA, USA). The system used Phenomenex BIOSEP-SEC 4000 column (500 A, 5 µm, 7.8 × 300 mm) (Waters Corporation Milford, MA, USA) in an acetonitrile buffer (50% acetonitrile and 0.1% (*w/v*) trifluoroacetic acid) with a running time of 10 min (2 mL/min flow rate). The column temperature was maintained at 25 °C and sample temperature at 15 °C. Injection volume was 50 µL, and UV-detection was done at 214 nm. The quantitative ratio of glutenins and gliadins was calculated according to Larroque et al. [41] by dividing the total amount of soluble and insoluble glutenins by the total amount of soluble and insoluble gliadins. The chromatogram for SE-HPLC was provided in the supplementary file (Figure S1).

Reverse-phase high-performance liquid chromatography (RP-HPLC) was used to determine the quantitative ratio of glutenin subunits (HMW/LMW) and the quantitative ratio of different gliadin fractions in total gliadins. For the analysis, 100 mg of the flour was used, and every sample was analyzed in triplicate. Gliadins were extracted by adding 1 mL of 70% (*v/v*) ethanol into tubes with flour; the tubes were vortexed and placed into the 60 °C water bath for 30 min followed by centrifugation for 5 min. The supernatant was filtered on a 0.45 μm PVDF filter, and gliadins were separated into three fractions: alfa + beta, omega and gamma gliadins. The precipitate with glutenin polymers was then twice flushed with 1 mL 50% (*v/v*) propan-1-ol, stored in a 60 °C water bath for 30 min and centrifuged at maximal speed. The supernatant was removed, and the remaining part was reduced in a buffer (50% (*v/v*) propan-1-ol, 2 M urea and 0.2 M Tris-HCl, pH 6.6) containing 1% (*w/v*) dithiothreitol and stored in a 60 °C water bath for one hour. Samples were then alkylated with 4-vinylpyridine, stored in a water bath for 15 min and centrifuged. After centrifugation, the supernatant was filtered on a 0.45 µm PVDF filter. Analyses were performed by The Waters Alliance™ HPLC System (Waters Corporation Milford, MA, USA) with 2695 Separation Unit and 2996 photodiode array detector. Glutenin subunits and gliadins were separated on a Supercosil LC-18 column (5 µm, 25 cm × 2.1 mm, 100 a) (Waters Corporation Milford, MA, USA). The column temperature was maintained at 60 °C while the sample was maintained at a temperature of 15 °C. Injection volume was 50 µL with a running time of 60 min (1 mL/min flow rate). Proteins were detected at 210 nm. The quantitative ratio of glutenin subunits (HMW/LMW) was calculated according to Marchylo et al. [42], and the chromatogram for RP-HPLC provided in the supplementary file (Figure S2). The quantitative ratio of different gliadin fractions was calculated by dividing the individual gliadin fraction by total gliadin fractions and the chromatogram provided in the supplementary file (Figure S3).

### 2.4. Statistical Analyses

Descriptive statistics were presented by box-and-whisker plots. Linear mixed model analysis (using the REstricted Maximum Likelihood algorithm, REML) was carried out using SPSS 16.0 software (SPSS Inc., Chicago, IL, USA) based on Virk et al. [43] to determine the variance components for protein content and composition contributed to the genotype, the environment and the genotype by environment interaction for spelt and bread wheat. The three growing sites (Hungarian conventional and organic site and Serbian conventional site) were regarded as different environments (E) for all the genotypes (G). The environmental and genotypic variances and variance of the genotype by environment interaction (G × E) were evaluated for each trait under both management systems (organic, conventional).

Analysis of variance (ANOVA) and Tukey’s post hoc test was applied to analyze and test differences between mean values of genotypes and environments. Principal component analysis (PCA) was used to explore patterns of relationships between genotypes and visualize their clustering based on the quality parameters of the grain and flour and the quantitative ratios of gluten proteins, glutenins and gliadins. Correlations between the traits for spelt and bread wheat species were determined with the Pearson’s correlation coefficient.

## 3. Results

### 3.1. Effect of Wheat Species, Growing Site and Field Management on Processing Quality

**Figure 1:**
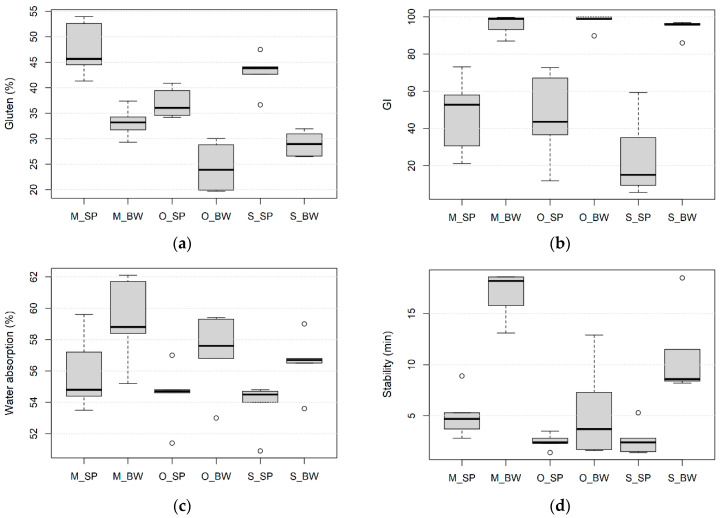

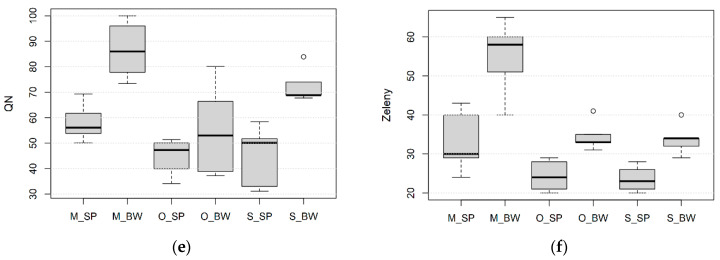
Boxplots for (**a**) gluten content (**b**), GI—gluten index (**c**), water absorption (**d**), dough stability time (**e**), QN—farinograph quality number (**f**)—Zeleny sedimentation volume; SP—spelt wheat, BW—bread wheat, M—conventional growing site in Martonvásár Hungary, O—organic growing site in Hungary, S—conventional growing site in Serbia; minimum value excluding outliers—shown by the line at the end of the bottom whisker; the first lower quartile (25th percentile)—shown by the lower end of the box; median—shown by the horizontal line inside the box; the third upper quartile (75th percentile)—shown by the upper end of the box; maximum value excluding outliers—shown by the line at the end of the upper whisker; outliers—shown by the empty circles.

Differences between wheat species and environmental conditions were found for gluten content, gluten index (GI), water absorption, dough stability, farinograph quality number (QN) and sedimentation volume (Figure 1).

Spelt wheat tended to have higher wet gluten content under both production system/three sites than bread wheat (Figure 1a). In addition, wet gluten content was generally higher at conventional sites than in the organic site. Wet gluten content ranged from 34.2% to 53.9%, with an average of 42.5% for spelt wheat, and from 19.7% to 37.4% with an average of 28.9% for bread wheat. The highest and the lowest gluten content were detected in spelt wheat at the conventional site in Hungary and in bread wheat at the organic growing site, respectively. The average value of GI was notably higher in bread wheat varieties (95.8) than in spelt varieties (39.5) in all three environments (Figure 1b). The variability of GI was more pronounced among spelt wheat than among bread wheat varieties. This parameter ranged from 9.4 to 67.2 for spelt and from 86.0 to 100 for bread wheat. No differences were observed among bread wheat varieties from three different growing sites, whereas for the spelt wheat, the varieties under conventional conditions in Serbia had lower GI than varieties at the organic site in Hungary.

The average values of flour water absorption were lower for spelt (3.6) than for bread wheat (11.1) in all environments (Figure 1c). This trait varied from 50.9 to 59.6 for spelt and from 53.0 to 62.1 for bread wheat. For both wheat species, the highest average value of flour water absorption was determined at the conventional growing site in Hungary, followed by the organic and conventional growing site in Serbia.

Dough stability time ranged from 1.4 min in spelt originated from the organic growing site to 18.6 min in bread wheat from a conventional growing site from Hungary (Figure 1d). The average values for dough stability time were 3.58 min for spelt and 11.1 min for bread wheat. The organic and conventional growing sites in Martonvásár, Hungary, had the lowest and the highest values of dough stability time for both bread and spelt wheat, respectively.

The QN of the bread wheat genotypes was higher (71.5) than that of spelt wheat (49.4). The highest QN was observed in bread wheat varieties from a conventional growing site in Hungary (86.6), followed by conventional growing sites in Serbia (72.6) and organic sites in Hungary (55.1) (Figure 1e).

The average value of the sedimentation volume, measured by the Zeleny test, was notably higher in bread wheat than in spelt wheat in all examined environments (Figure 1f). The highest values of sedimentation values for both species were determined for varieties grown at the conventional growing site in Hungary, while the sedimentation values at the organic site of Hungary and the conventional site of Serbia were similar both for spelt and for bread wheat.

### 3.2. Effect of Wheat Species, Growing Site and Field Management on the Kernel Size, Protein Content and Composition

**Figure 2:**
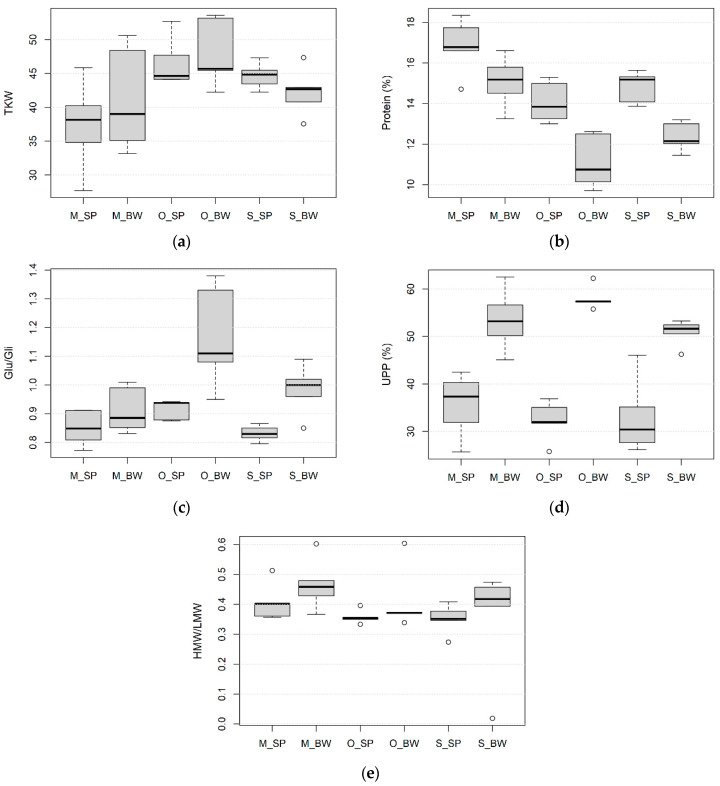
Boxplots for (**a**) TKW—thousand-kernel weight, (**b**) protein content, (**c**) Glu/Gli—the quantitative ratio of glutenins to gliadins, (**d**) UPP%—the percentage of unextractable polymeric proteins, (**e**) HMW/LMW—the ratio of high molecular weight and low molecular weight gluten subunits; SP—spelt wheat, BW—bread wheat, M—conventional growing site in Martonvásár, Hungary, O—organic growing site in Hungary, S—conventional growing site in Serbia; minimum value excluding outliers—shown by the line at the end of the bottom whisker; the first lower quartile (25th percentile)—shown by the lower end of the box; median—shown by the horizontal line inside the box; the third upper quartile (75th percentile)—shown by the upper end of the box; maximum value excluding outliers—shown by the line at the end of the upper whisker; outliers—shown by the empty circles.

The size of the kernel generally has a significant effect on the compositional traits of the kernels. The TKW ranged from 27.7 g to 52.7 g for spelt and from 33.2 g to 53.6 g for bread wheat, with the average values of 42.9 g and 43.8 g for spelt and bread wheat, respectively (Figure 2a). Bread wheat varieties had slightly higher TKW than spelt wheat at the conventional and organic production in Hungary. On the other hand, spelt varieties had, on average, remarkably higher TKW than the bread wheat varieties under the conventional site of Serbia. The highest TKW was measured under organic conditions, while the lowest TKW was observed at the conventional site in Hungary for both bread and spelt wheat. The variation of this trait was the largest at the conventional site in Martonvásár, Hungary.

The percentage of total protein content was statistically higher in spelt wheat than in bread wheat varieties at all growing sites, with the average values of 15.2% and 12.9%, respectively (Figure 2b). It ranged from 11.47% to 15.97%. Genotypes of both spelt and bread wheat from the conventional growing site in Hungary had the highest protein content, followed by the conventional growing site in Serbia, while the varieties from the organic growing site had the lowest protein content (Table 4).

Glutenin to gliadin ratio (Glu/Gli) was on average higher in bread wheat than in spelt wheat (Figure 2c). In bread wheat, Glu/Gli varied from 0.75 to 1.29, while in spelt wheat, it varied from 0.80 to 0.97. The highest Glu/Gli values for both wheat species were determined at the organic site in Hungary. Differences between Glu/Gli at the conventional sites in Hungary and Serbia were not significant for spelt. In bread wheat, Glu/Gli was significantly higher at the conventional site in Serbia than in Hungary (Table 4).

A higher average value of UPP was measured in bread wheat varieties (53.4%) than in spelt (33%) (Figure 2d). UPP ranged from 25.7% to 46.6% in spelt and from 39.3% to 65.8% in bread wheat. The growing sites for spelt wheat varieties did not significantly differ in UPP. However, in bread wheat, differences were found only between the conventional site in Serbia and the organic site in Hungary (Table 4).

The quantitative ratio of the high and the low molecular weight glutenin subunits statistically differed between the management systems and two species (Figure 2e and Table 4). The HMW/LMW values were, on average higher for bread (0.44) than for spelt (0.37) wheat. The HMW/LMW ranged from 0.40 to 0.55 in bread and from 0.33 to 0.42 in spelt wheat. Under conventional growing conditions, these parameters were higher in Hungary than in Serbia for both species. For bread wheat, HMW/LMW was significantly higher at the conventional sites than at the organic site, while for spelt wheat, differences between conventional growing sites from Serbia and organic growing sites were not significant.

**Figure 3:**
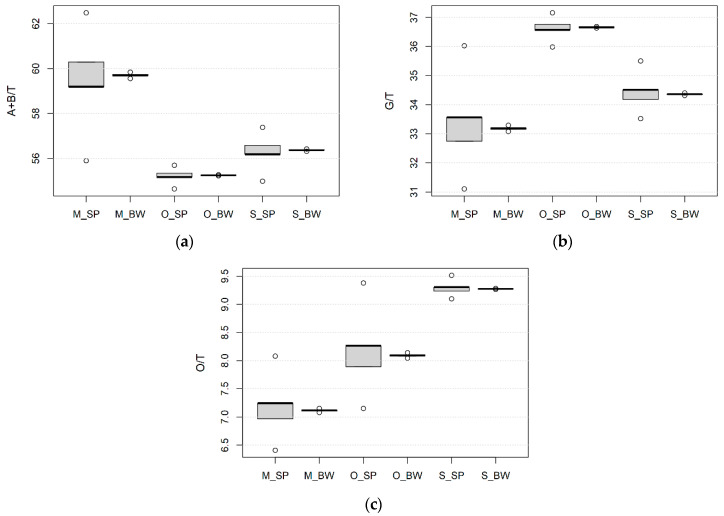
Boxplots for (**a**), A+B/T—the quantitative ratio of alfa and beta gliadins in total gliadins, (**b**) G/T—the quantitative ratio of gamma gliadins in total gliadins, (**c**) O/T—the quantitative ratio of omega gliadins in total gliadins; SP—spelt wheat, BW—bread wheat, M—conventional growing site in Martonvásár, Hungary, O—organic growing site in Hungary, S—conventional growing site in Serbia; minimum value excluding outliers—shown by the line at the end of the bottom whisker; the first lower quartile (25th percentile)—shown by the lower end of the box; median—shown by the horizontal line inside the box; the third upper quartile (75th percentile)—shown by the upper end of the box; maximum value excluding outliers—shown by the line at the end of the upper whisker; outliers—shown by the empty circles.

The quantitative ratio of alfa and beta gliadins in total gliadins (A+B/T) ranged from 53.76 to 58.70. No differences between spelt and bread wheat were observed for A+B/T (Table 4). The quantitative ratio of different gliadin fractions in total gliadins varied among the environments (Figure 3a–c). For both species, A+B/T was the highest at the conventional site in Hungary, followed by the conventional site in Serbia and the organic site in Hungary. The ratio of gamma in total gliadins (G/T) ranged from 30.31 to 37.65. It was higher in spelt wheat (35.23) than in bread wheat (32.76). The G/T at the organic site in Hungary was the highest for spelt and bread wheat, while this parameter was the lowest at the conventional site in Hungary. The omega gliadins in total gliadins (O/T) varied from 7.53 to 14.29 and were higher in bread (11.31) than in spelt (8.04) wheat. The highest and the lowest O/T were at the conventional site in Serbia and Hungary, respectively.

Analysis of variance showed significant differences between the genotypes, environment and G × E interactions for total protein content and composition (Table 4). Among the spelt varieties, the highest protein content was determined for Ostro and Oberkulmer-Rotkorn (15.90% and 15.97%, respectively), and the lowest for Schwabenkorn (14.34%). The protein content was the highest in Pobeda (13.56%) bread wheat varieties, while Apache had the lowest protein content (11.47%). The spelt varieties Rouquin and Ostro had the highest and the lowest values, respectively, for Glu/Gli and UPP. Among the bread wheat varieties, the highest Glu/Gli was found in Apache (1.29). UPP did not vary significantly among the bread wheat varieties. Among the spelt varieties, HMW/LMW was the highest in Baulander Spelz (0.42), while among the bread wheat varieties, the highest HMW/LMW was measured in Balkan (0.55). The increased A+B/T was observed in spelt varieties Ostro (58.52) and Oberkulmer-Rotkorn (58.70) and in bread wheat varieties Pobeda (58.13) and Recital (58.32). The G/T was the highest in Rouquin (37.65) spelt wheat and in Estevan (35.70) bread wheat. Balkan variety had the highest O/T (14.29), whereas, among the spelt wheat, the highest O/T was determined in Baulander Spelz (8.99) and Rouquin (8.48). Differences among the genotypes, growing sites (E), and G × E interactions were significant for all traits, except for the G × E for Glu/Gli and E for UPP in spelt wheat, and for G and G × E for UPP in bread wheat.

### 3.3. Differences in the Genotype and Environmental Effects on Spelt and Bread Wheat Properties

**Figure 4:**
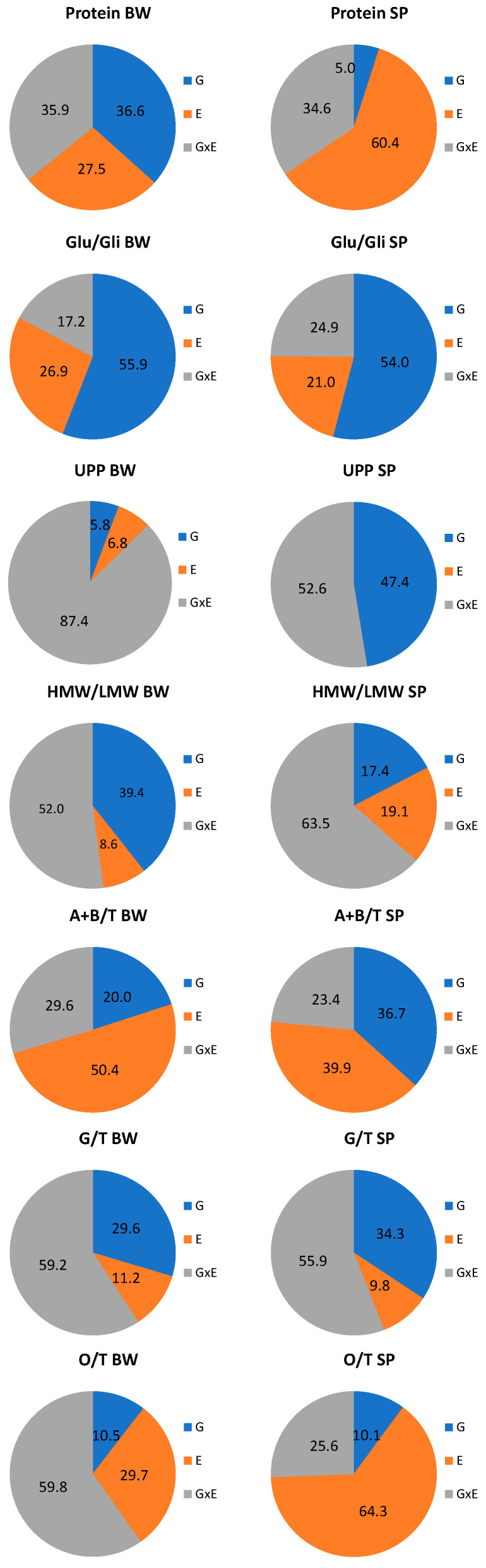
Pie diagrams of the relative contribution of genotype (G), environment (E—3 growing sites), genotype × environment interaction (G × E) to the total sum of squares for the protein content and composition for spelt (SP) and bread wheat (BW), PC—total protein content, Glu/Gli—the quantitative ratio of glutenins to gliadins, UPP%—unextractable polymeric proteins, HMW/LMW—the quantitative ratio of high molecular weight and low molecular weight glutenin subunits, A+B/T—the portion of alfa and beta gliadins in total gliadins, G/T—the portion of gamma gliadins in total gliadins, O/T—the portion of omega gliadins in total gliadins.

Variance component analysis showed that the effect of the variety (genotype) was the greatest for Glu/Gli in bread and spelt wheat, accounting for more than 50% of the total variability and was also considerable for UPP in spelt wheat (Figure 4). The greatest effect of the environment (E, growing site) was observed for A+B/T in bread and spelt wheat (50% and 40%, respectively), O/T and the protein content in spelt wheat (>60%). The contribution of genotype × environment interaction was the most pronounced for UPP in bread wheat (>80%) and spelt (>50%), HMW/LMW and G/T in both species (>50%), and O/T in bread wheat (>50%).

### 3.4. Principal Component Analysis of Spelt and Bread Wheat Grown under Different Field Managements and in Different Countries

**Figure 5:**
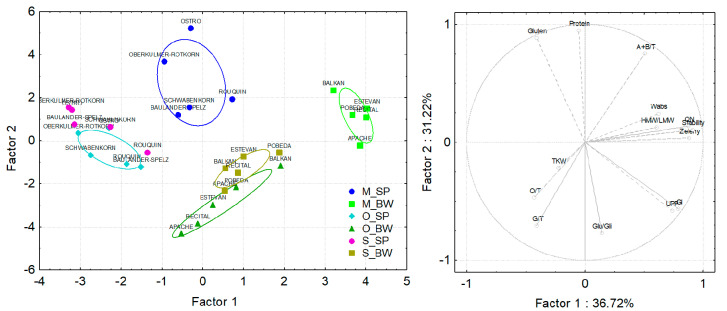
Principal component analysis of spelt and bread wheat varieties based on all analyzed compositional and functional quality traits. M—conventional growing site in Martonvásár, Hungary, O—organic growing site in Hungary, S—conventional growing site in Serbia. SP—spelt wheat, BW—bread wheat. GI—gluten index, Wabs—water absorption, Stability—dough stability time, QN—farinograph quality number, Zeleny—Zeleny sedimentation volume, TKW—thousand-kernel weight, Glu/Gli—the quantitative ratio of glutenins to gliadins, UPP—unextractable polymeric proteins, HMW/LMW—the quantitative ratio of high molecular weight and low molecular weight glutenin subunits, A+B/T—the portion of alfa and beta gliadins in total gliadins, G/T—the portion of gamma gliadins in total gliadins, O/T—the portion of omega gliadins in total gliadins.

The PCA, based on combining all the analyzed compositional and processing quality traits, clearly distinguished the conventionally grown bread and spelt wheat varieties in Hungary from the other groups the fewer extent varieties from the organic site in Hungary and the conventional site in Serbia (Figure 5). The first PC accounted for 36.72% of the total variation and was determined by water absorption, dough stability, sedimentation volume and HMW/LMW. The bread varieties from the conventional site in Hungary with high values of these parameters were clustered on the right side of the biplot. On the opposite side of the biplot were positioned the spelt wheat varieties from the organic site in Hungary and the conventional site in Serbia. The PC2 accounted for 31.22% of the total variation and was determined by gluten and protein content, Glu/Gli and G/T. The bread wheat varieties from organic locations in Hungary with high Glu/Gli and G/T grouped near the Glu/Gli and G/T vectors, opposite from the high gluten and protein spelt wheat varieties from the conventional location in Hungary.

### 3.5. Correlations of Compositional and Processing Quality Traits in Spelt and Bread Wheat

**Figure 6:**
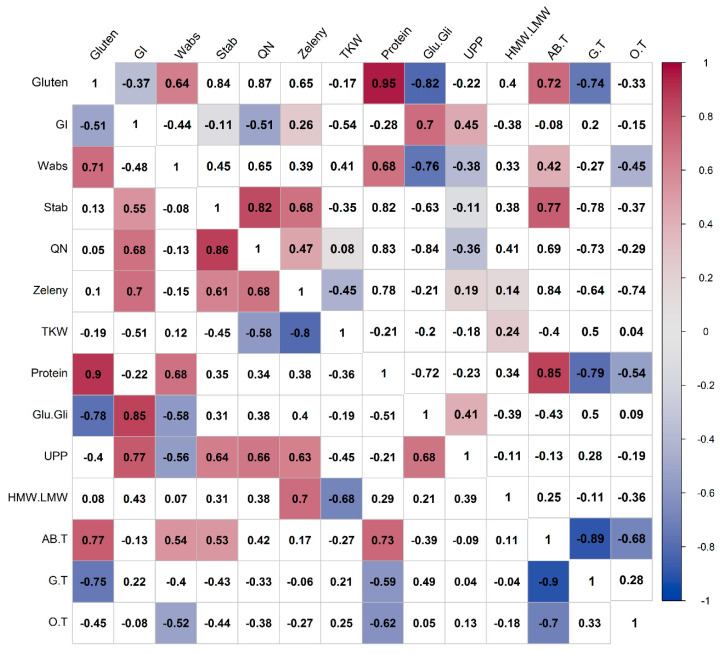
Correlation coefficients between the processing quality, protein content and composition for bread (above the diagonal) and spelt (below the diagonal) wheat. GI—gluten index, Wabs—water absorption, Stab—dough stability, QN—farinograph quality number, Zeleny—sedimentation volume, TKW—thousand-kernel weight, Glu.Gli—the quantitative ratio of glutenins to gliadins, UPP—unextractable polymeric proteins, HMW.LMW—the quantitative ratio of high molecular weight and low molecular weight glutenin subunits, AB.T—the portion of alfa and beta gliadins in total gliadins, G.T—the portion of gamma gliadins in total gliadins, O.T—the portion of omega gliadins in total gliadins. The values that are highlighted are correlation coefficients significant for *p* < 0.05. The values in red and blue cells show significant positive and negative correlations, respectively. The correlation coefficients that are not highlighted (in white cells) are not significant.

Pearson’s coefficients showed significant positive correlations between gluten content and water absorption, dough stability, QN, sedimentation volume, protein content and A+B/T in bread wheat (Figure 6). In spelt wheat, gluten content was positively correlated with fewer traits, namely water absorption, protein content and A+B/T. Significant negative correlations were determined between gluten content and Glu/Gli and G/T in both species, and also between gluten content and GI in spelt wheat. QN was in positive correlations with GI, sedimentation volume and UPP in spelt wheat. In bread wheat, QN was in positive correlations with water absorption, protein content and A+B/T, while in both species, QN was found to be positively correlated with dough stability.

Positive correlations were found between protein content and gluten content, water absorption, dough stability, sedimentation volume, QN and A+B/T in bread wheat. Protein content was negatively correlated with Glu/Gli, G/T and O/T in both bread and spelt wheat varieties. Glu/Gli was positively related to GI and UPP in both species. Negative correlations were found between Glu/Gli and gluten content, water absorption, dough stability, QN and protein content in bread wheat, while the negative correlations were observed only between Glu/Gli and gluten, water absorption and protein content in spelt wheat. The UPP showed significant positive correlations with GI, dough stability, QN, sedimentation volume and Glu/Gli, and negative correlations with water absorption in spelt wheat. A positive correlation was observed between HMW/LMW and sedimentation volume, while a negative correlation was found between HMW/LMW and TKW in spelt wheat. TKW was also insignificant negative correlations with GI in bread and spelt wheat and with QN and sedimentation volume in spelt wheat. The A+B/T was positively correlated with gluten and protein content and dough stability in both species, with QN and sedimentation volume in bread wheat, and with water absorption in spelt wheat. Negative correlations were found between A+B/T and G/T and O/T in both spelt and bread wheat. The G/T and O/T were negatively correlated with most of the quality traits.

## 4. Discussion

### 4.1. Differences between Spelt and Bread Wheat on Processing Quality, Protein Content and Composition

An irreplaceable role of wheat in the daily diet and increasing interest in consuming organically grown food due to its beneficial effect on the environment and human health advanced the research on the grain and flour quality parameters, protein content and composition of spelt and bread wheat grown under different management systems [17]. The spelt wheat varieties were characterized by higher average values for protein and wet gluten content and lower GI, sedimentation volume, water absorption and dough stability than bread wheat varieties. The considerably higher protein and wet gluten content determined in spelt were corroborated by other studies [44,45,46,47]. A high positive correlation between these two quality parameters in spelt wheat in our study confirmed the finding of Rapp et al. [48]. Moreover, the higher GI and sedimentation volume found in bread wheat varieties were in agreement with previous findings [47,49,50]. Spelt wheat had less favorable rheological properties than bread wheat, such as lower water absorption and shorter dough stability, which were also determined by other authors [5,7,51]. The lower water absorption in spelt wheat, despite higher protein content, could be explained by the differences in kernel hardness between spelt and bread wheat. The analyzed spelt varieties were predominately soft [52,53], while most bread wheat varieties had hard endosperm texture [54,55]. Hardness index was found to be in positive correlation with water absorption [56] as higher starch damage during the milling process, and large particle size in hard wheat increase the water absorption [57]. The water absorption and stability time were positively related to farinograph QN, as was confirmed in previous studies [58,59]. These two quality traits are important as their lower values could negatively affect the elasticity and extensibility of dough and consequently gave smaller loaf volume [5,60]. A significant positive correlation between sedimentation volume and protein content in bread wheat was also found by Aydogan et al. [58]. Significant positive correlations between water absorption and protein content, wet gluten and QN and between protein and wet gluten content in bread wheat in our study are in agreement with Denčić et al. [59].

The compositional traits are generally highly related to the size of the kernels, which also determines the yield of the grain and the flour [61]. The average values of the TKW for spelt and bread wheat in our study did not differ. Similar results with no significant difference for TKW between spelt and bread wheat were obtained from Zaneti et al. [62]. In contrast, Petrenko et al. [51] and Markowski et al. [63] reported higher TKW for spelt than for bread wheat. It is possible that the lack of significant differences in TKW between spelt and bread wheat in our study could be attributed to the large variation of this trait among the analyzed genotypes. Moreover, if we consider only the Serbian site where the variation of TKW is much less than in Hungary, spelt varieties had considerably higher TKW than bread wheat varieties. The differences in spike morphology between these two species can be explained by genetic factors, mainly by the Q gene [64]. A longer spike and fewer grains per spikelet in spelt could provide more space for the determination of more endosperm cells during the grain differentiation phase in favorable environmental conditions and, subsequently, the formation of larger grains. Nevertheless, TKW and seed morphology is the characteristic of the specie, and the effect of the spike architecture on the size of the kernels should be considered within each species.

Processing quality traits depend on the quantity and quality of gluten proteins, such as the relative amount of monomeric to polymeric gluten proteins (gliadins to glutenins ratio), HMW-GS to LMW-GS and the amount of UPP [65]. According to ANOVA, the protein content and composition showed significant differences between genotypes and wheat species. Higher Glu/Gli, HMW/LMW and UPP were found in the bread wheat varieties than in spelt wheat varieties, similarly to Koehler et al. [66], who found a lower reverse ratio Gli/Glu in bread than in spelt wheat. The Glu/Gli defines the balance between elastic and viscous properties of wheat dough [11]. Call et al. [67] also measured higher HMW/LMW in bread than in spelt wheat. Significantly higher UPP in bread wheat than spelt wheat was also found by Hussain et al. [68], while no significant differences were identified for Glu/Gli or LMW/HMW-GS in another study [69]. The viscoelastic properties of gluten matrix depend on the qualitative and quantitative balance of polymeric glutenins, responsible for intermolecular disulfide linkages and monomeric gliadins, responsible for intramolecular linkages [70]. In wheat grain or flour, the main building blocks of the gluten protein matrix are HMW polymeric glutenins that form its backbone, while LMW glutenin subunits extend and terminate gluten chain structure. The gluten polymer size and complexity are measured by the percentage of UPP, which contributes to good gluten strength and baking performance [70,71]. In gluten matrix, gliadins interact with other proteins only non-covalently and are therefore less effective in influencing viscoelastic properties and breadmaking quality [72]. The prevalence of monomeric gliadins in relation to the polymeric glutenins, a small portion of UPP and lower HMW/LMW resulted in less favorable viscoelastic properties of the spelt gluten matrix. Thus, the spelt dough is characterized by weaker gluten structure [13], lower stability and elasticity, and higher extensibility than bread wheat [73], being soft and sticky and forming small loaves [62].

Zhang et al. [74] found a significantly positive correlation between sedimentation volume and the HMW/LMW in bread wheat. Positive correlations between these two traits were significant only in spelt wheat in our study. The GI was positively correlated with Glu/Gli, as was also confirmed by Edwards et al. [75]. Correlation analysis showed negative relations between the protein content and the Glu/Gli in both species, which was in agreement with the findings of Dhaka [76]. In our study, positive correlations were calculated between UPP and quality traits (i.e., GI, dough stability, QN and sedimentation volume) in spelt wheat only. The UPP was in positive correlations with dough stability [71] and GI [77] in bread wheat and with sedimentation volume [78] and GI [75] in durum wheat. The positive relationship found between the UPP and Glu/Gli in spelt wheat in this study could be explained by the fact that glutenins are polymeric proteins.

The average values of A+B/T for spelt and bread wheat genotypes did not significantly differ. The reason could be that spelt, and bread wheat have 98.5% of identity in an amino acid sequence of an alfa type gliadin [79]. Hence the diversity between species is smaller than the diversity within the species [80]. The spelt wheat varieties had on average higher G/T and lower O/T than the bread wheat. This is in accordance with the previous electrophoretic analyses of glutenins pattern, which showed the absence of the omega gliadins bands in spelt wheat comparing to bread wheat [81,82]. However, Podolska et al. [50] detected a higher amount of all three glutenin fractions in bread than in spelt wheat. Significantly lower HMM/LMW and higher G/T in spelt wheat in comparison to bread wheat in our study could be explained by the similarity in molecular weight, amino acid composition and nucleic acid sequence between omega gliadins and LMW glutenin subunits [83,84]. We determined positive correlations of A+B/T with most of the quality traits in spelt and bread wheat. Similarly, positive relations between the gliadins and sedimentation volume, an important indicator of good breadmaking performance was found by Sozinov and Poperelya [85]. In this study, all significant correlations between G/T and O/T with quality traits were negative, although more negative correlations with quality traits were determined for G/T than for O/T. Results of many studies on the effect of gliadins on rheological properties of the dough and end-use quality are inconclusive. The good quality of bread wheat was associated with gamma gliadin [86,87] and omega gliadin fraction, which were negatively associated with the bread quality [86,88]. Nevertheless, Khatkar et al. [72] reported that all three fractions of gliadins significantly improved its breadmaking performance.

### 4.2. The Effect of the Genotype and the Outstanding Characteristics of the Varieties

The effect of the genotype, the growing site (environment) and genotype × environment interaction was observed on the variation of all protein content and composition traits, but their degree depended on the wheat species and the specific trait in question. Numerous studies determined the genotype [74,75,89,90], environment [91] and G × E interaction [48,59,81] before as important factors that contribute significantly to the variability of wheat quality traits. In addition to the genetic control of the quantity and composition of wheat gluten [12], environmental conditions can also cause significant variation in the amount and composition of the gluten proteins [92,93,94].

Based on the variance components calculated by linear mixed model analysis, the greatest effect of the genotype was found on the Glu/Gli both in spelt and bread wheat, as regards to the compositional traits of the kernels. Furthermore, protein content, HMW/LMW and G/T were also significantly determined by the genotype in bread wheat. In spelt, however, the UPP, A+B/T and G/T were the most significantly determined by the genotype. Relatively high values of broad-sense heritability for glutenins (0.78), gliadins (0.60), Glu/Gli (0.81), and UPP (0.63–0.65) determined in previous studies [95,96] imply the possibility of an effective selection in the breeding programs for these quality traits. The effect of the genotypes on all traits was significant for both spelt and bread wheat, except on the UPP, for which no significant differences were observed among bread wheat varieties, which may be due to less genetic variation present in the analyzed genotypes.

The growing interest in organic crop production raised the need for developing varieties with specific traits suitable for organic low input agriculture, such as better nutrient-efficiency and grain quality [97]. Modern wheat varieties usually have lower nitrogen and protein content under organic conditions [98]. The N availability for organic crops could be additionally restricted due to reduced microbial mineralization activity under unfavorable conditions [99]. Therefore, better nitrogen use efficiency is crucial for wheat production with restricted inputs, such as organic farming. Grain protein content could be used as a simple indicator for the nitrogen efficiency of a crop [97]. Based on the PCA results, the spelt varieties Ostro and Oberkulmer-Rotkorn, and the bread wheat varieties Balkan, Estevan and Pobeda, were characterized with the highest protein content, which makes them suitable for low input and organic management systems with limited nitrogen application. Similar to our findings, Ostro had the highest protein content in comparison to other spelt wheat varieties [5], while Oberkulmer-Rotkorn had the higher protein content among six varieties [100] and was the second-ranked among the 12 spelt genotypes [101]. Bread wheat variety Pobeda was characterized by good quality parameters determined in other studies [102,103,104] that corroborated our results.

Among the spelt varieties, Schwabenkorn and Roquin had the highest Glu/Gli and UPP. In the study of Schober and Kuhn [105], Schwabenkorn was the only pure spelt variety grouped with modern bread wheat according to the baking quality. Our results showed that the spelt variety Rouquin was distinct from the other genotypes by the high values of traits that are responsible for good breadmaking quality, namely Glu/Gli, UPP, HMW/LMW, O/T and G/T. This could be due to the contribution of bread wheat in the Rouquin pedigree. The variety Rouquin was developed from a cross between the Swedish bread wheat variety Virtus with the Belgium spelt wheat Lignée 24, which was then back-crossed to Lignée 24 and then crossed with Swiss spelt variety Altgold [105]. In our study, Rouquin had the highest UPP value among the spelt genotypes, which could be attributed to 5 + 10 HMW-GS in *Glu-D1* locus found in previous research [29,106]. Likewise, Rouquin, bread wheat variety Estevan had high average values for most of the traits, namely protein content, Glu/Gli, UPP and G/T. These two varieties could be a valuable source of enhanced protein composition properties.

Considering the possible positive effects of gliadin fractions on rheological parameters and bread quality, as well as their negative implications on the immunopathogenesis of coeliac disease, the quantification of each gliadin fraction in different wheat varieties could be important for discriminating genotypes with extreme contents of alfa, beta, gamma and omega gliadins, either to improve wheat quality parameters or to identify genotypes with reduced gliadin fractions that are responsible for gluten intolerance.

To date, gliadins fraction patterns of 400 Spanish spelt genotypes with polyacrylamide gel electrophoresis [107] and electropherograms of 27 European spelt varieties [105] were obtained to elucidate genetic diversity and to facilitate cultivar identification. In previous research, quantification of gliadins fractions was done to compare the quantities of gliadin fractions between different wheat species [9,50,108,109], using only one spelt wheat genotype in each study. Rodríguez-Quijano et al. [110] found considerable differences in the amounts of different gliadin fractions between two spelt varieties, with one of them having more similar values to bread wheat varieties. To the best of our knowledge, until now, no research was conducted to investigate the quantitative variation of gliadin fractions in more spelt genotypes and between different environments. In our work, among five analyzed spelt varieties, Ostro and Oberkulmer-Rotkorn were characterized with the highest A+B/T and the lowest O/T and G/T, while Rouquin had the lowest A+B/T and the highest O/T and G/T. The significant differences in gliadin content between the analyzed varieties were corroborated with the previously observed great diversity of gliadin patterns that could be used for differentiating wheat varieties [111]. We found no genotype with either a high or low ratio for all gliadin fractions due to the negative correlations between A+B/T and O/T and G/T. Nevertheless, the presence of genetic variability in the quantity of gliadin fractions suggests the possibility of developing low and high gliadin fraction varieties by break the genetic linkage using advanced molecular and conventional breeding tools.

### 4.3. The Effect of Environment on Processing Quality, Protein Content and Composition of Spelt and Bread Wheat Varieties

In this study, the effect of the different field management systems and different countries were evaluated on the processing quality, protein content and composition of spelt and bread wheat varieties.

The field management affected the physical properties of the kernels. The highest values of the TKW were found under the organic management system for both species, which is in agreement with the findings of Ingver et al. for bread wheat [112]. Nevertheless, other studies [14,113] had opposite findings, while Mazzoncini et al. [23] and Fares et al. [114] found no significant differences in the TKW of the conventionally and organically grown hexaploid and tetraploid wheat. Consequently, the results found at this time are contradictory.

Higher values of total protein and wet gluten content, sedimentation volume, QN and dough stability were observed both for bread and spelt wheat at the conventional growing site. Higher total protein content under conventional conditions comparing to organic production was also found by others [23,113]. These differences could be the result of the higher N input applied through fertilizers at the conventional site and the lack of application of N fertilizers at the organic site. However, the Hungarian conventional site differed more from the Hungarian organic site than the Serbian conventional site. Differences in analyzed traits between the conventional sites in Hungary and Serbia could be due to differences between agronomic practices and previous crops between the two countries (Table 2). The increased protein content may also be associated with the appropriate doses and timing of the N application applied by fertilizers [115], resulting in higher humus and total nitrogen content in the soil [116].

Similarly, differences in O/T between Serbia and Hungary could be attributed to different nutrient availability in soil, as was reported by Dupont et al. [117]. Furthermore, the different edaphic and meteorological conditions in the two countries could also have an influence on the trait variations. Although geographically close, growing sites could considerably vary in microclimatic and soil conditions causing larger variation of certain traits [118]. Some quality parameters, like gluten index and sedimentation volume, were considerably lower in Serbia than in Hungary. Meteorological conditions during the last 100 days before harvest in Serbia were characterized by a higher amount of cumulative precipitation and mean temperatures (Table 3). Moreover, the precipitation in May during anthesis at the Serbian site was 240% higher, and the temperature was 2.6 °C lower than the long-term average. Such an abundant moisture regime could significantly reduce gluten index, possibly by the accumulation of storage proteins in an unbalanced ratio [119]. Both environmental and genetic factors interfering the plant development before anthesis could be responsible for modifying the protein structure through starch-protein accumulation interrelations [120]. The grain-filling stage was marked with a dry period and heat stress in June and the beginning of July, with a mean temperature of 3.4 °C higher than the long-term average. Vida et al. [121] determined a moderate but significant effect of high-temperature at the end of the grain-filling stage on the reduction of gluten index. An increase in temperature was shown to decrease the percentage of unextractable polymeric protein in total polymeric protein (%UPP), indicating either a decreased polymer size or complexity [120].

The difference that appeared in the quality traits of the conventional and organic sites may also result from the changes that appeared in the composition of the protein due to the different field management practices. Notably, lower HMW/LMW and A+B/T and higher G/T and O/T were typical at the organic site compared to the conventional site. Most of these findings were supported by other studies [23,112,113,114,122] except that we have found no difference in the gluten index, while other studies did [123].

Flour protein content and the protein composition determined in our study were found to be affected by environmental fluctuations, in both species, except for UPP in spelt wheat. It seems that the strong genotypic variations, independent of environments, are predominant for UPP in spelt wheat. The significant positive influence of the environment and the lack of significance of G × E interaction on UPP reported in our study are in agreement with previous findings [75].

Wheat processing quality traits and protein content and composition differed in their response to environmental conditions and growing sites. Based on the variance components calculated by the linear mixed model analysis, the greatest relative contribution of the growing site (E) was found on protein content in spelt wheat, A+B/T in bread and spelt wheat, and on O/T in spelt wheat. This suggests that these traits may be important for use in selection in the targeted environments.

The considerable effect of the G × E interaction was also found in this study for Glu/Gli, UPP, HMW/LMW and G/T. Significant effect of G × E, including management practices effect, were determined for HMW/LMW in bread wheat by Triboi et al. [124]. The information of the influence of G × E interaction on quality and protein processing traits is important for wheat breeders as the considerable interaction could contribute to hampering the selection process when genotype performance varies across the testing environments, but equally to a better choice of varieties for quality enhancement for certain environments.

## 5. Conclusions

The results of this study are somewhat limited since they were based on one-year field trials in Serbia and Hungary, including a single organic and two conventional sites. However, they clearly distinguished spelt from bread wheat, the organic from conventional sites and, moreover, the differences among the varieties for each species. Protein content and composition of spelt and bread wheat significantly differed, except for the A+B/T, indicating the better processing quality traits of the bread wheat. The spelt varieties contained more gluten and protein. The latter was generally related to the increased gliadin content contributing to the higher extensibility of the dough, which was rather specific to spelt wheat. Bread wheat varieties were characterized with better breadmaking potential than spelt wheat, having a significantly higher amount of glutenins, UPP and HMW/LMW. Greater kernel size (TKW), lower HMW/LMW, lower alfa+beta gliadin content (A+B/T), and higher gamma gliadin (G/T) content were typical at the organic site compared to the conventional site, resulting in a generally better processing quality of seeds grown at the conventional sites. The spelt varieties Ostro and Oberkulmer-Rotkorn and the bread wheat varieties Balkan, Estevan and Pobeda were found to be the most suitable for low input and organic management systems, being most tolerant to limited nitrogen application.

## Figures and Tables

**Table 1:** List of analyzed spelt and bread wheat varieties, including the country of origin, the year of release, the high molecular weight glutenin subunits (HMW-GS) allelic composition and the Glu-scores according to the literature data.

Species/Variety	Pedigree	Origin	Year	HMW-GS			Glu-Score	Ref.
				Glu-A1	Glu-B1	Glu-D1		
*T. aestivum* L. subsp. *spelta*								
Baulander Spelz	Geiberger Spelz	DEU	1926	1	13 + 16	2 + 12	8	[29,30,31,32]
Ostro ^†^	Oberkulmer-Rotkorn/Steins-Roter-Tiroler	CHE	1978	1	6 + 8	2 + 12	6	[29,30]
				2 *	6 + 8	2 + 12	6	[33]
Rouquin ^†^	Lignée-24/Ardenne//Altgol	BEL	1979	1	6 + 8	2 + 12	6	[33]
				1	6 + 8	5 + 10	8	[29]
Schwabenkorn	(S)LV	DEU	1988	1	6 + 8	2 + 12	6	[29,30,33]
Oberkulmer-Rotkorn	(S)LV-CHE	CHE	1948	1	6 + 8	2 + 12	6	[29]
*T. aestivum* L. subsp. *aestivum*								
Apache	Axial/NRPB-84-4233	FRA	1998	N	7 + 9	2 + 12	5	[34]
Balkan	Bačka/Bez1//Miron808/3/NS433/4/Skor35	SRB	1979	2 *	7 + 9	5 + 10	9	[35]
Estevan	Capo/SE-24090	DEU	2009	1	7 + 9	5 + 10	9	[36]
Pobeda	Sremica/Balkan	SRB	1990	2 *	7 + 9	5 + 10	9	[37]
Recital	Mexique-267(R-267)/9369	FRA	1986	2 *	6 + 8	5 + 10	8	[38]
				2 *	7 + 9	5 + 10	9	[30]

^†^ The biotype of the spelt variety Ostro with Glu-A1. 2 * subunit and the biotype of the spelt variety Rouquin with Glu-D1 5 + 10 subunit were used for analyses in this study. BEL—Belgium; CHE—Switzerland; DEU—Germany; FRA—France; SRB—Serbia, Glu—glutenin.

**Table 2:** Growing and environmental conditions of different management systems in Hungary and Serbia.

Growing Conditions		Hungary	Serbia
Location	geographic coordinates	47°18′ N, 18°47′ E	45°20′ N, 19°51′ E
	Altitude	115 m	84 m
Growing parameters	previous crop: conventional organic	oil radish phacelia	soybean
	sowing density	550 seeds/m^2^	550 seeds/m^2^
Soil parameters	soil type	chernozem	chernozem
	pH (KCl)	7.25	7.41
	humus (*m/m*%)	2.8	2.6
	P_2_O_5_ (mg/kg)	210	208
	K_2_O (mg/kg)	210	176
	yearly average nitrogen input through nitrogen, phosphorus and potassium (NPK) combined fertilizer (conventional) (active ingredient, kg/ha)	120	100

**Table 3:** Meteorological conditions for the 2018/2019 growing season in Hungary and Serbia.

		Hungary	Serbia
full season	Growing period (days)	279	264
	Cumulative precipitation (mm)	365.6	435.3
	Mean temperature (°C)	9.3	11.5
	Absolute min temperature (°C)	−14.4	−16.3
	Absolute max temperature (°C)	36.0	35.0
last 100 days	Cum. precipitation in the last 100 days before harvest (mm)	225.0	265.4
	Mean temperature in the last 100 days (°C)	17.1	17.5
	Absolute min temp in the last 100 days (°C)	−0.7	−0.9
	Absolute max temp in the last 100 days (°C)	36.0	35.0
abs. min-max	No of days with Tmin ≤ 0 °C	90	75
	No of days with Tmin ≤ −10 °C	6	3
	No of days with Tmax ≥ 25 °C	42	38
	No of days with Tmax ≥ 30 °C	16	17
	No of days with Tmax ≥ 35 °C	1	1

**Table 4:** Total protein content and gluten proteins content depending on the wheat variety and growing site.

Variety/Growing Site	Protein (%)	Glu/Gli	UPP (%)	HMW/LMW	A+B/T	G/T	O/T
*T. aestivum* L. subsp. *spelta*							
Baulander Spelz	15.22 ^c^	0.88 ^b,c^	34.87 ^b,c^	0.42 ^c^	55.17 ^b^	35.84 ^b^	8.99 ^b^
Ostro	15.90 ^d^	0.80 ^a^	25.87 ^a^	0.38 ^b,c^	58.52 ^d^	33.93 ^a^	7.55 ^a^
Rouquin	14.77 ^b^	0.97 ^d^	38.00 ^c^	0.38 ^b,c^	53.96 ^a^	37.65 ^c^	8.48 ^a,b^
Schwabenkorn	14.34 ^a^	0.90 ^c,d^	35.81 ^b,c^	0.35 ^a,b^	57.36 ^c^	34.97 ^b^	7.67 ^a^
Oberkulmer-Rotkorn	15.97 ^d^	0.82 ^a,b^	30.43 ^a,b^	0.33 ^a^	58.70 ^d^	33.76 ^a^	7.53 ^a^
Average	15.2 ^B^	0.87 ^A^	33.00 ^A^	0.37 ^A^	56.74 ^A^	35.23 ^B^	8.04 ^A^
M	16.83 ^c^	0.87 ^a^	33.62 ^a^	0.41 ^b^	59.02 ^c^	34.40 ^a^	6.58 ^a^
O	14.07 ^a^	0.92 ^b^	32.28 ^a^	0.36 ^a^	54.89 ^a^	36.30 ^b^	8.81 ^b^
S	14.81 ^b^	0.84 ^a^	33.09 ^a^	0.35 ^a^	56.32 ^b^	34.93 ^a^	8.74 ^b^
Genotype (G)	<0.0001 ***	<0.0001	0.0002 ***	0.0001 ***	<0.0001 ***	<0.0001 ***	0.0004 ***
Growing site (E)	<0.0001 ***	0.0012 **	0.7744 n.s.	0.0002 ***	<0.0001 ***	<0.0001 ***	<0.0001 ***
G × E	<0.0001 ***	0.1282 n.s.	0.0383 *	0.0005 ***	<0.0001 ***	<0.0001 ***	0.0094 **
*T. aestivum* L. subsp. *aestivum*							
Apache	11.47 ^a^	1.29 ^d^	56.90 ^a^	0.42 ^a,b^	53.76 ^a^	33.91 ^b,c^	12.33 ^c^
Balkan	13.48 ^c^	0.75 ^a^	51.89 ^a^	0.55 ^c^	55.40 ^b^	30.31 ^a^	14.29 ^d^
Estevan	13.52 ^c^	1.00 ^b^	56.45 ^a^	0.41 ^a^	54.03 ^a^	35.70 ^c^	10.26 ^b^
Pobeda	13.56 ^c^	0.96 ^b^	48.94 ^a^	0.40 ^a^	58.13 ^c^	32.96 ^b^	8.91 ^a^
Recital	12.26 ^b^	1.12 ^c^	53.02 ^a^	0.43 ^b^	58.32 ^c^	30.93 ^a^	10.75 ^b^
Average	12.9 ^A^	1.02 ^B^	53.44 ^B^	0.44 ^B^	55.93 ^A^	32.76 ^A^	11.31 ^B^
M	15.07 ^c^	0.91 ^a^	53.50 ^a,b^	0.47 ^c^	58.51 ^c^	31.38 ^a^	9.71 ^a^
O	11.15 ^a^	1.18 ^c^	58.01 ^b^	0.41 ^a^	52.63 ^a^	33.27 ^b^	10.11 ^a^
S	12.36 ^b^	0.99 ^b^	48.81 ^a^	0.44 ^b^	56.64 ^b^	33.64 ^b^	14.10 ^b^
Genotype (G)	<0.0001 ***	<0.0001 ***	0.2297 n.s.	<0.0001 ***	<0.0001 ***	<0.0001 ***	<0.0001 ***
Growing site (E)	<0.0001 ***	<0.0001 ***	0.0157 *	<0.0001 ***	<0.0001 ***	<0.0001 ***	<0.0001 ***
G × E	<0.0001 ***	<0.0001 ***	0.1947 n.s.	<0.0001 ***	<0.0001 ***	0.0002 ***	<0.0001 ***

Protein—total protein content (%), Glu/Gli—the quantitative ratio of glutenins to gliadins, UPP—unextractable polymeric proteins (%), HMW/LMW—the quantitative ratio of high molecular weight and low molecular weight glutenin subunits, A+B/T—the portion of alfa and beta gliadins in total gliadins, G/T—the portion of gamma gliadins in total gliadins, O/T—the portion of omega gliadins in total gliadins; M—conventional growing site in Martonvásár, Hungary, O—organic growing site from Hungary, S—conventional growing site from Serbia, ***—*p* < 0.001, **—*p* < 0.01, *—*p* < 0.05, n.s.—not significant. Different upper case letters indicate significant differences (*p* < 0.05) between average values of spelt and bread wheat. Means followed by a common lower case letter are not significantly different (*p* < 0.05) within each species.

## Data Availability

Data is contained within the article.

## Supplementary Materials

The following are available online at https://www.mdpi.com/2304-8158/10/1/156/s1, Figure S1: The chromatogram for SE-HPLC analysis representing individual peaks for total wheat proteins, encompassing soluble and insoluble proteins, Figure S2: The chromatogram for RP-HPLC analysis representing the individual peaks for HMW and LMW glutenin subunits, Figure S3: The chromatogram for RP-HPLC analysis representing the individual peaks for ω, α + β and γ gliadins.
