# Supplementary material for: Differences in Processing Quality Traits, Protein Content and Composition between Spelt and Bread Wheat Genotypes Grown under Conventional and Organic Production
Source: Foods. 2021 Jan 13;10(1):156. doi: 10.3390/foods10010156 (PMC7828489; doi:10.3390/foods10010156)
Supplement: Supplementary file 1 [file foods-10-00156-s001.zip › Suplementary figures.pdf]

Supplementary

Differences in processing quality traits, protein content and composition between spelt and bread wheat genotypes grown under conventional and organic production

Verica Takač[1]*, Viola Tóth[2], Marianna Rakszegi[2], Sanja Mikić[1], Milan Mirosavljević[1] and Ankica Kondić-Špika[1]

[1] Institute of Field and Vegetable Crops, Maksima Gorkog 30, 21000 Novi Sad, Serbia; verica.takac@ifvcns.ns.ac.rs (V.T.); sanja.mikic@ifvcns.ns.ac.rs (S.M.); milan.mirosavljevic@ifvcns.ns.ac.rs (M.M.); ankica.spika@ifvcns.ns.ac.rs (A.K.S.)
[2] Agricultural Institute, Centre for Agricultural Research, Brunszvik u. 2, 2462-Martonvásár, Hungary; rakszegi.mariann@atk.hu (M.R.); toth.viola@atk.hu (T.V.)
* Correspondence: verica.takac@ifvcns.ns.ac.rs; Tel.: +381-21-4898-214
Received: date; Accepted: date; Published: date

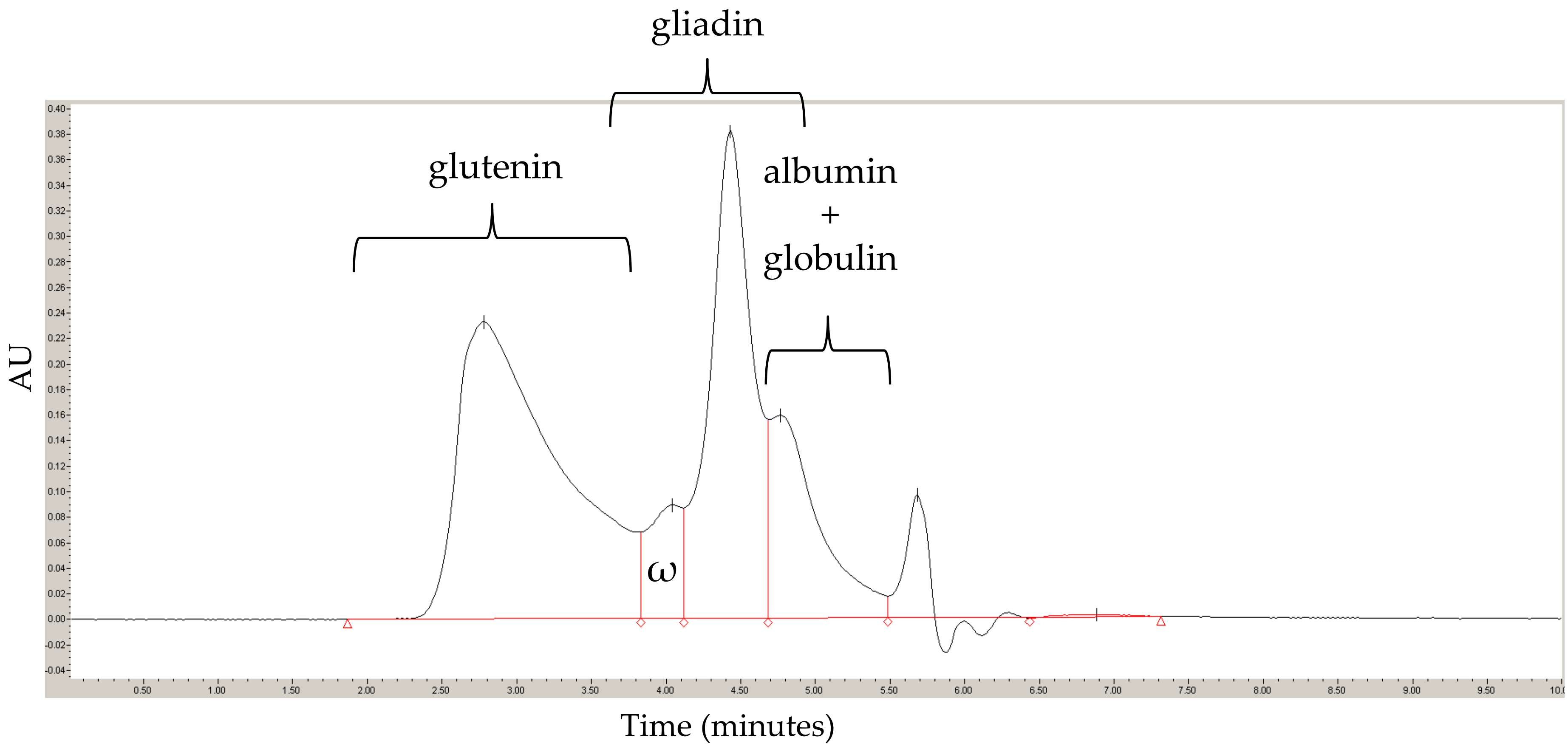

Figure S1. The chromatogram for SE-HPLC analysis representing individual peaks for total wheat proteins, encompasing soluble and insoluble proteins

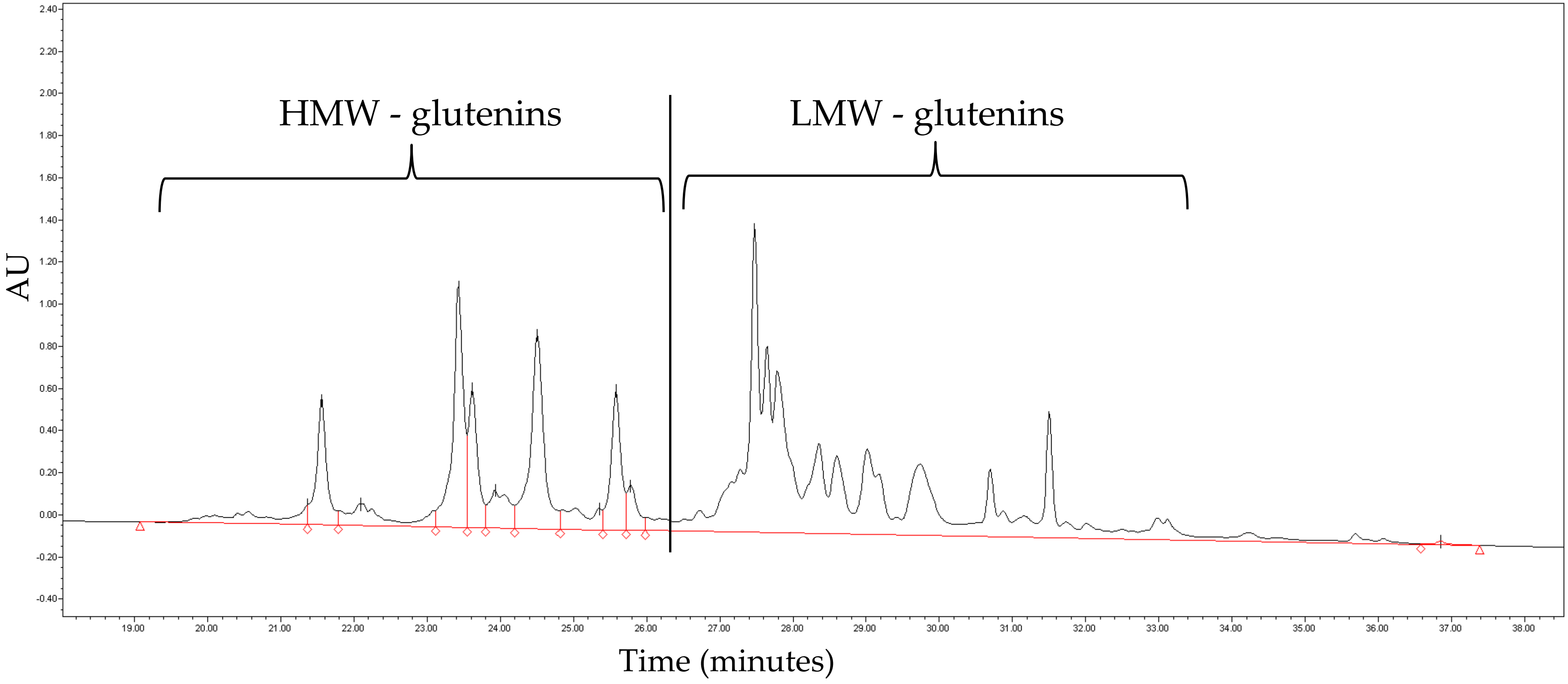

Figure S2. The chromatogram for RP-HPLC analysis representing the individual peaks for HMW and LMW glutenin subunits

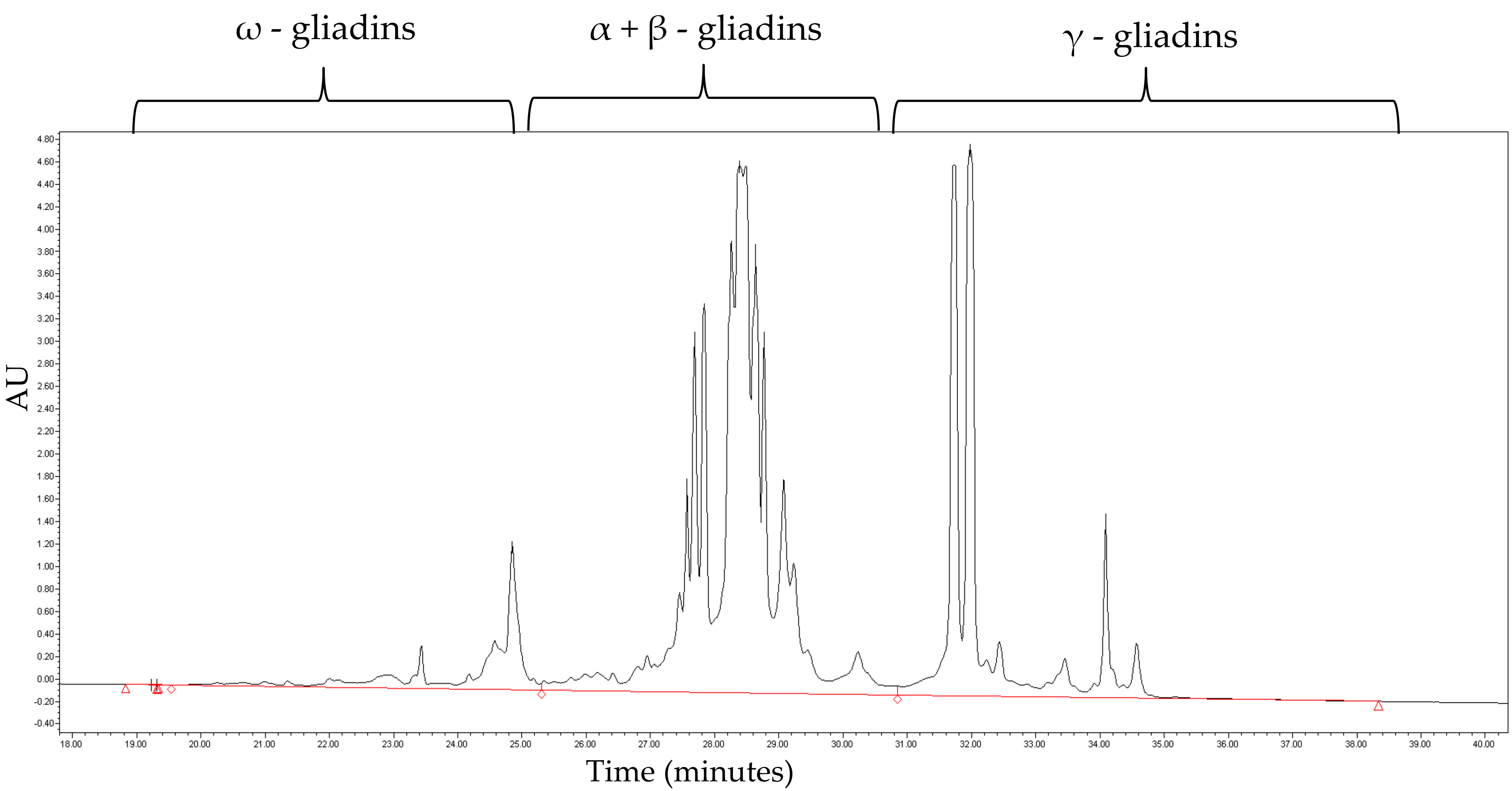

Figure S3. The chromatogram for RP-HPLC analysis representing the individual peaks for ω, α + β and γ gliadins
